# An Intelligent Non-Invasive Blood Pressure Monitoring System Based on a Novel Polyvinylidene Fluoride Piezoelectric Thin Film

**DOI:** 10.3390/mi15050659

**Published:** 2024-05-17

**Authors:** Shilin Li, Taoyun Zhou, Muzhou Liu, Qiaomei Zhao, Yi Liu

**Affiliations:** School of Information, Hunan University of Humanities, Science and Technology, Loudi 417000, China

**Keywords:** polyvinylidene fluoride (PVDF), flexible pressure sensors, intelligent blood pressure monitoring

## Abstract

Hypertension is a common cause of cardiovascular diseases, closely associated with the high mortality and disability rates of cardiovascular diseases such as stroke and coronary heart disease. Therefore, developing a comfortable and sustainable device for monitoring human pulse signals holds practical significance for the prevention and treatment of hypertension and cardiovascular diseases. PVDF flexible pressure sensors possess the characteristics of high sensitivity, good flexibility, and strong biocompatibility, thereby demonstrating extensive application potential in areas such as health monitoring, wearable devices, and electronic skins. This paper focuses on the development of a modified piezoelectric polymer and its application in an intelligent blood pressure monitoring system, demonstrating its outstanding performance and feasibility through a series of experiments. This research provides innovative material choices for the development of intelligent medical devices and offers beneficial guidance for the design and application of future intelligent health monitoring systems.

## 1. Introduction

With the development of society and the improvement of people’s living standards, health has become one of the key concerns. Blood pressure, as a crucial indicator of physical health, holds significant importance in preventing chronic diseases, particularly cardiovascular diseases. Cardiovascular diseases are common in both China and globally, serving as a major factor contributing to the rise in global mortality and disability rates [1]. High blood pressure is a common trigger for cardiovascular diseases with high mortality and disability rates, such as stroke and coronary heart disease. Therefore, there is a critical need for heightened awareness of hypertension and the adoption of effective preventive and therapeutic measures [2]. In 2019, global statistics from the World Health Organization revealed that there were 1.13 billion hypertensive patients, with 7.5 million deaths attributed to high blood pressure, accounting for 12.8% of global mortality. Hypertension, as a widespread global disease, not only prevails among the middle-aged and elderly populations but also exhibits a trend toward affecting younger individuals. However, only 54% of hypertensive patients receive effective treatment and successfully control their conditions. Consequently, achieving continuous blood pressure monitoring and early diagnosis of cardiovascular diseases has become an urgent issue that needs to be addressed.

Abundant clinical data indicate a close correlation between the amplitude, frequency, and characteristic points of pulse waves and cardiovascular diseases [3]. By examining the relationship between pulse waves and various parameters of the cardiovascular system, it is possible to assess the health status of the human cardiovascular system [4]. Therefore, developing a device that is comfortable and capable of continuously monitoring human pulse signals holds practical significance for the prevention and treatment of diseases such as hypertension and cardiovascular ailments.

## 2. Related Work

In recent years, scholars both domestically and internationally have extensively researched pulse detection devices, primarily focusing on pressure-based sensors and optoelectronic sensors [5]. Pressure sensors convert external pressure signals into readable signals such as resistance, voltage, and capacitance, enabling the detection of external pressure. There are extensive applications for the Internet of Things, consumer electronics, and healthcare fields [6]. Traditional pressure sensors are primarily composed of rigid materials like metal and semiconductors, offering advantages such as low cost and broad applicability. However, they suffer from issues like poor consistency and stability. With the advancement of technology, the increasing demands for product applications have rendered conventional rigid pressure sensors inadequate. Consequently, sensor technology is evolving towards flexibility, miniaturization, and intelligence, penetrating deeply into various aspects of everyday life.

**Figure 1:**
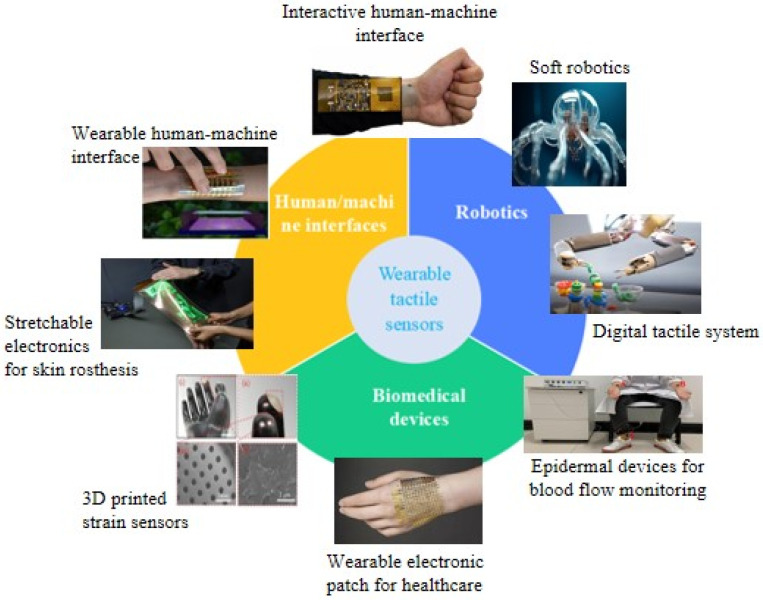
Related applications of flexible pressure sensors.

As an emerging pressure-sensitive component, flexible pressure sensors possess perception capabilities similar to human skin, offering vast application prospects in health monitoring [7], electronic skin [8], intelligent robots [9], and other fields; related applications of flexible pressure sensors are shown in Figure 1. They undergo deformation under external force, altering the electrical properties of the sensitive material and converting pressure signals into electrical signals, thereby achieving the detection of complex parameters. Nano-conductive materials, including metal nanoparticles, metal nanowires, conductive carbon black, graphene, and single-walled carbon nanotubes (SWCNTs), are commonly used as sensitive layers in flexible pressure sensors [10]. According to their operating principles, flexible pressure sensors are primarily classified into three types: capacitive, resistive, and piezoelectric.

Capacitive flexible pressure sensors typically consist of electrodes, a substrate, and a dielectric layer, resembling a parallel-plate capacitor. The working principle involves changes in the geometric structure of the electrodes and dielectric layer under external loads, altering the distance between the electrodes and plates, resulting in a change in capacitance. Li et al. [11] and others developed a highly sensitive capacitive flexible pressure sensor by utilizing a highly porous PDMS elastic dielectric layer and gaps inserted between the conductive polymer/electrode, capable of detecting pressure within a wide pressure range. Zhuo B [12] and colleagues used a microstructured PDMS as the dielectric layer to create a highly sensitive capacitive flexible pressure sensor. Its sensitivity under low-pressure conditions (<0.2 kPa) was 1.62 kPa^−1^, while under high-pressure conditions (>1 kPa), the sensitivity was 0.05 kPa^−1^.

Resistive-type flexible pressure sensors, based on the piezoresistive effect, can convert external pressure signals into resistance or current signals. In 2014, Zhu B [13] and others designed a resistive-type flexible pressure sensor based on a microstructured graphene array. The microstructured graphene film was directly attached to a flexible PET film with a conductive indium tin oxide coating. The pyramid microstructure of the graphene array not only imparted an extremely high sensitivity (5.5 kPa^−1^) within the low-pressure range (<100 Pa), more than 50 times that of non-microstructured sensors, but also exhibited excellent stability and a fast response time (0.2 ms). Wang X [14] and colleagues designed a resistive-type flexible pressure sensor consisting of two layers of SWCNT/PDMS film. They transferred microstructures from silk to prepare a PDMS microstructured film. The sensor demonstrated high sensitivity (1.80 kPa^−1^), enabling the detection of weak pressures, and featured fast response time (<10 ms), high stability (>67,500 cycles), and low detection limit (0.6 Pa).

Piezoelectric-type flexible pressure sensors, based on the piezoelectric effect, convert pressure signals into electrical signals. Piezoelectric materials undergo polarization under mechanical loads, separating positive and negative charges. This process results in equal and opposite charges on the upper and lower surfaces of the piezoelectric material, creating a potential difference. Piezoelectric-type flexible pressure sensors exhibit high sensitivity, instantaneous response, and a broad frequency response range (10 Hz to 50 kHz). They are suitable for dynamic signal measurements, utilizing the detection of the output electrical signal to achieve pressure measurements. Organic piezoelectric materials, such as polyvinylidene fluoride (PVDF) and its derivatives, as well as polylactic acid, are also known as piezoelectric polymers. They offer advantages such as good flexibility, impact resistance, stable performance, and a high piezoelectric constant, making them ideal materials for preparing flexible pressure sensors.

PVDF is a semi-crystalline, chemically inert thermoplastic fluoropolymer composed of long-chain molecules with a repeating unit of -[CF2-CH2]-, where hydrogen and fluorine atoms alternate. It has a molecular weight of approximately 10^5^ and consists of around 10^3^ repeating basic units. Due to the abundance of dipole moments along the polymer chain, PVDF exhibits excellent piezoelectric, ferroelectric, and pyroelectric properties. There are three common crystal structures of PVDF, known as the α-phase, β-phase, and γ-phase, consisting of trans (T) and gauche (G) conformations. At room temperature and pressure, the most easily obtained is the α-phase, where the molecular chain adopts a helical TGTG’ structure. In the α-phase, the dipole moments within cancel each other out, resulting in zero net dipole moment and low piezoelectric and ferroelectric properties. The β-phase, with a planar zigzag TTT structure, exhibits the best piezoelectric properties among the three crystalline forms. The fluorine atoms are located on the same side of the polymer chain, leading to strong spontaneous polarization with a polarization intensity of around 130 mC/m^2^, making it a commonly used crystal structure for flexible pressure sensors. The γ-phase is a crystalline form achieved through high-temperature or high-pressure treatment in organic solvents, characterized by a trans conformation; each single crystal cell contains two trans chains.

PVDF flexible pressure sensors, characterized by high sensitivity, excellent flexibility, and strong biocompatibility, exhibit extensive potential applications in health monitoring, wearable devices, and electronic skin. In 2015, Lei et al. [15] designed a PVDF sensor patch for monitoring respiratory signals during dynamic walking. In 2018, Sekine T and collaborators [16] utilized a polyvinylidene fluoride–trifluoroethylene copolymer (PVDF-TrFE) as the sensitive layer to develop a fully printed, wearable pulse sensor capable of monitoring pulse signals on the skin. In the same year, Gong Wang and co-workers [17] designed a flexible pressure sensor based on a PVDF nanofiber membrane, demonstrating excellent piezoelectric performance. Apart from detecting quantitative pressure signals, it can also monitor human motion signals in real time, successfully capturing subtle movement signals such as joint bending and vocal cord vibration.

In China, PVDF flexible pressure sensors find widespread applications in flexible wearables, health monitoring, and other fields. Ling Z B and collaborators [18] developed a real-time respiratory signal detection device based on PVDF piezoelectric film, consisting of PVDF piezoelectric film, an expandable belt, and elastic structures. The device successfully detected complete, smooth, and non-excessively saturated respiratory waveforms. Xu Z J and colleagues [19] utilized PVDF piezoelectric film as the sensitive unit to design a PVDF respiratory sensor, monitoring respiratory signals by detecting the bending deformation of the PVDF piezoelectric film under the influence of respiratory airflow. Chen et al. [20] used PVDF piezoelectric film to develop a heart rate sensor for measuring heart rate, overcoming drawbacks such as the inconvenience and restrictive force associated with traditional measurement methods. Xin Y and co-workers [21] created a sleep monitoring pillow based on a PVDF pressure sensor, capable of monitoring physiological indicators such as pulse, respiration, and snoring during sleep in real-time. It can also identify and alert to abnormal pulse and respiratory patterns, achieving functions like recognizing sleep apnea and automatically monitoring snoring.

Due to its advantages such as high piezoelectric constant, excellent mechanical properties, outstanding chemical stability, and remarkable flexibility, PVDF is currently one of the most researched piezoelectric polymers and a hot topic in the field of flexible pressure sensors. Traditional PVDF-based pressure sensors have sensitivities that meet general blood pressure monitoring requirements. However, for critically ill patients, even subtle changes can become crucial factors influencing the direction of treatment research. Therefore, enhancing the sensitivity of blood pressure monitoring devices remains one of the key technical challenges to be addressed. In this context, this paper proposes a novel piezoelectric polymer material by modifying PVDF, aiming to improve the sensitivity of the sensing material and thereby enhance the accuracy of blood pressure detection.

The key contributions of this paper can be summarized as (1) the successful modification of polyvinylidene fluoride (PVDF) by introducing polyvinyl alcohol, resulting in a novel piezoelectric polymer with outstanding piezoelectric performance and electromechanical coupling characteristics. This material demonstrates a more sensitive sensing ability in blood pressure monitoring systems, improving the accuracy of blood pressure monitoring. (2) The introduction of *N*,*N*-dimethylacetamide promotes the formation of the β-phase in PVDF films, enhancing the piezoelectric coefficients and sensitivity while simplifying the manufacturing process. The incorporation of *N*,*N*-dimethylacetamide lowers the glass transition temperature, enhancing flexibility and wearability. (3) Branching polyvinyl alcohol with citric acid increases the dielectric constant of the piezoelectric polymer, enhancing charge storage capacity, stability, and reliability. Citric acid branching of polyvinyl alcohol also accelerates the degradation rate of PVDF, improving environmental friendliness and sustainability.

The paper is organized as follows. Section 2 provides an academic review of relevant studies. Section 3 outlines the design and optimization of the intelligent non-invasive blood pressure monitoring system. Experiments and simulations in Section 4 demonstrate the enhanced accuracy of the blood pressure monitoring system in this paper. Conclusions are summarized in Section 5.

## 3. Design and Optimization of the Non-Invasive Blood Pressure Monitoring System

**Figure 2:**
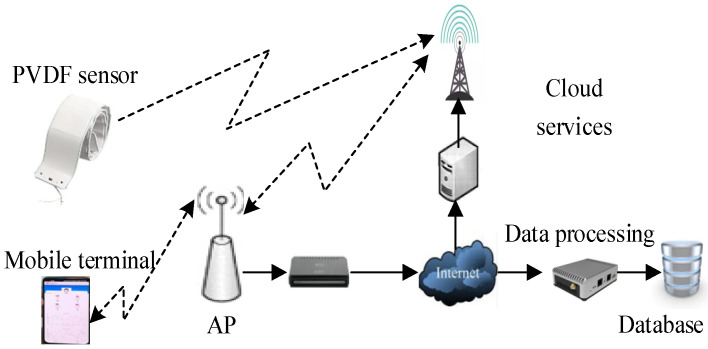
The schematic diagram of the blood pressure monitoring system based on PVDF sensors.

The intelligent non-invasive blood pressure monitoring system based on modified piezoelectric polymers is illustrated in Figure 2, employing a modular design. The entire system consists of three main components: the polyvinylidene fluoride (PVDF) sensor, handheld mobile terminal, and cloud service center. The PVDF sensor utilizes a modified piezoelectric polymer as its sensitive material and establishes a connection with the handheld terminal through Bluetooth technology. The system facilitates mutual communication between control nodes via the mobile terminal and wearable display device, achieving the reception of the pulse wave data, waveform display, and calculation of blood pressure values. Simultaneously, the system transmits monitoring data to a remote medical service monitoring platform through the mobile network. Powered by a compact polymer lithium battery, the non-invasive blood pressure monitoring system is equipped with a display function for convenient real-time observation, enabling the real-time or continuous display of processed data obtained from the mobile terminal.

### 3.1. A Novel Highly Sensitive PVDF Sensor Technology

PVDF is a novel polymeric piezoelectric material with outstanding piezoelectric effects. In comparison to traditional piezoelectric materials, PVDF film exhibits superior characteristics such as broad frequency response, large dynamic range, and high sensitivity in electromechanical conversion. The output voltage of the PVDF film is linearly proportional to the applied external force. When the film undergoes mechanical deformation due to external forces, the generated charge remains in a linear relationship with the applied force conditions.

#### 3.1.1. Innovative Modified Piezoelectric Polymer Materials

In this study, polyvinyl alcohol is used to modify polyvinylidene fluoride (PVDF), resulting in the successful preparation of a piezoelectric polymer with outstanding piezoelectric performance and electromechanical coupling characteristics. When applied in a blood pressure monitoring system, this novel piezoelectric polymer can sensitively detect changes in blood pressure, thereby enhancing the accuracy of blood pressure monitoring. Its main components include polyvinylidene fluoride, polyvinyl alcohol, and *N,N*-dimethylacetamide, prepared in a weight ratio of 66:21:450. The piezoelectric polymer exists in the form of a polyvinylidene fluoride solution and a polyvinylidene fluoride film.

The preparation process of polyvinylidene fluoride (PVDF) solution is as follows:

(1) Add polyvinylidene fluoride, polyvinyl alcohol, and *N*,*N*-dimethylacetamide to a container in a weight ratio of (61–71):(19–22):(400–500). Stir the mixture uniformly at 60–80 °C for 2–4 h to ensure thorough mixing.
(2) Maintain the temperature of the mixed solution, let it stand for 4–6 min to remove air bubbles, and obtain the PVDF solution.

The preparation process of polyvinylidene fluoride (PVDF) film is as follows:

(1) Add polyvinylidene fluoride, polyvinyl alcohol, and *N*,*N*-dimethylacetamide to a container in a weight ratio of (61–71):(19–22):(400–500). Stir the mixture uniformly at 60–80 °C for 2–4 h to obtain a casting solution.
(2) Maintain the temperature of the casting solution and let it stand for 4–6 min to remove air bubbles. Pour the solution onto a glass plate to form a uniform coating with a thickness of 0.8–1.2 mm.
(3) Immerse the glass plate with the coating into deionized water at 25–28 °C. Once the coating detaches from the glass plate, soak it in deionized water for 45–50 h to form the PVDF film. After removing the film, air-dry it at 25–28 °C.

Polyvinyl alcohol is a hydroxyl-rich polymer with strong hydrophilicity and polarity. In the preparation process of the polyvinyl alcohol piezoelectric polymer, at the microscopic level, it exhibits intertwining between polyvinyl alcohol and polyvinylidene fluoride, the formation of hydrogen bonds, and interactions involving strong Van der Waals forces, ultimately forming a supramolecular composite. The blending or grafting process of polyvinyl alcohol and polyvinylidene fluoride introduces hydroxyl groups onto the polyvinylidene fluoride molecular chains. These hydroxyl groups can form hydrogen bonds with water, enhancing the polarity and hydrophilicity of polyvinylidene fluoride.

Polyvinyl alcohol piezoelectric polymer helps reduce the crystallinity and glass transition temperature of polyvinylidene fluoride, thereby enhancing its flexibility and processability. When this polymer is prepared as films or fibers, it forms a porous structure, increasing the surface area of polyvinylidene fluoride and the contact area with water, thereby improving its hydrophilicity. This piezoelectric polymer can generate electrical signals when subjected to pressure changes.

In addition, the introduction of *N*,*N*-dimethylacetamide promotes the formation of the β-phase in polyvinylidene fluoride films, enhancing the piezoelectric coefficient and sensitivity. *N*,*N*-dimethylacetamide forms strong interactions with polyvinylidene fluoride molecules through hydrogen bonding and dipole–dipole interactions, stabilizing the β-phase structure and preventing its transformation into the α-phase. The β-phase is a polar crystalline form of polyvinylidene fluoride, exhibiting excellent piezoelectric performance and electromechanical coupling characteristics. In contrast, the α-phase is a nonpolar crystalline form with inferior piezoelectric and electromechanical coupling properties.

*N*,*N*-dimethylacetamide can also lower the glass transition temperature of polyvinylidene fluoride films, enhancing their flexibility and wearability. As a polar, non-protonic solvent, *N*,*N*-dimethylacetamide can dissolve polyvinylidene fluoride, forming a homogeneous solution. When the solution solidifies into a film under certain conditions, *N*,*N*-dimethylacetamide remains between the polyvinylidene fluoride molecular chains, acting as a plasticizer to reduce intermolecular forces and lower the glass transition temperature of polyvinylidene fluoride. Additionally, the introduction of *N*,*N*-dimethylacetamide can improve the sustainability and environmental friendliness of polyvinylidene fluoride. The composite film formed by *N*,*N*-dimethylacetamide and polyvinylidene fluoride exhibits advantages such as improved hydrophilicity, resistance to pollution, and biocompatibility.

In more detail, *N*,*N*-dimethylacetamide is a small molecule. After introducing *N*,*N*-dimethylacetamide into the polymer system, it can separate the segments of polyvinylidene fluoride and weaken the intermolecular forces between polyvinylidene fluoride molecular chains, making them more prone to movement, thereby enhancing the plasticity of polyvinylidene fluoride. *N*,*N*-dimethylacetamide molecules insert themselves between the polyvinylidene fluoride molecular chains, alleviating the stress between these chains, making the polyvinylidene fluoride molecular chains more mobile, reducing their crystallinity, and thereby increasing the plasticity of polyvinylidene fluoride. These changes counteract the factors that limit the plasticity of polyvinylidene fluoride, including the crystallization and stress of polyvinylidene fluoride molecular chains.

#### 3.1.2. Citric Acid-Guided Polyvinyl Alcohol Branching

The polyvinyl alcohol used in this paper undergoes citric acid-guided treatment, and the preparation method is as follows: mix polyvinyl alcohol and citric acid in a weight ratio of 1: (0.8–1.2), react evenly at 60–80 °C for 1–3 h, and stir continuously during the reaction. Afterward, cooling and crystallization yield citric acid-guided polyvinyl alcohol.

Citric acid-guided polyvinyl alcohol is a water-soluble polymer material that can be blended with polyvinylidene fluoride (PVDF) to form a composite film with improved hydrophilicity, anti-pollution properties, and biocompatibility. It should be noted that using citric acid-guided polyvinyl alcohol in PVDF piezoelectric polymer can increase the dielectric constant of PVDF but does not enhance its piezoelectric coefficient. This is because citric acid-guided polyvinyl alcohol is mainly distributed in the amorphous region of PVDF, which primarily consists of the amorphous phase of PVDF and may even disrupt the crystallization of the β-phase of PVDF. Therefore, the introduction of citric acid-guided polyvinyl alcohol does not induce the formation of the β-phase in PVDF, and consequently, it does not enhance its piezoelectric effect. However, the increased dielectric constant contributes to storing more charge under the action of an electric field, improving dielectric strength and breakdown field strength, thereby providing higher stability and reliability.

### 3.2. Principle of Pulse Monitoring Based on Piezoelectric Polymers

#### 3.2.1. Method of Connecting Piezoelectric Polymers

PVDF piezoelectric film possesses advantages such as a wide frequency response range, high sensitivity, light weight, flexibility without brittleness, impact resistance, resistance to water, and chemical contamination. Moreover, it can be shaped arbitrarily, making it highly suitable for manufacturing wearable sensors. The sensor comprises a flexible substrate and conductive electrodes, with a piezoelectric polymer overlaid on the flexible substrate. This piezoelectric polymer can be configured on the flexible substrate in two ways. The first method involves attaching a polyvinylidene fluoride (PVDF) film, stretched uniaxially, on one side of the flexible substrate, with both sides of the PVDF film connected to the conductive electrodes. The second method entails coating the flexible substrate with a solution of PVDF, followed by heat treatment, and similarly connecting both sides of the flexible substrate to the conductive electrodes. The designed blood pressure monitoring device takes the form of a bar-shaped structure worn on the wrist. The PVDF pressure sensor within it can adapt to the curvature of the wrist, covering the position of the artery to meet the requirements of blood pressure monitoring.

#### 3.2.2. Blood Pressure Monitoring Principle Based on PVDF Sensors

After uniaxial stretching, the polyvinylidene fluoride film is pasted onto one side of the flexible substrate, and both sides of the polyvinylidene fluoride film are connected to conductive electrodes to form a pressure sensor, which is attached to the wrist and electrically connected to the signal processing system.

The PVDF pressure sensor generates a small amount of charge under external loads and is prone to leakage. The high sensitivity of piezoelectric thin films results in poor electromagnetic interference resistance of sensors, making them susceptible to noise interference and distortion. Therefore, in order to facilitate subsequent data collection and analysis, it is necessary to use a signal conditioning circuit to convert and amplify the weak charge signal output. The signal conditioning circuit designed in this article consists of a charge amplification circuit and a low-pass filtering circuit. The previous charge amplification circuit is used to convert and amplify charge signals into voltage signals and to enhance the high impedance of PVDF pressure sensors and convert input to low-impedance output. The low-pass filtering circuit in the later stage is used to suppress and remove noise that is significantly higher than the frequency of the pulse signal during the data acquisition process.

The signal processed by the circuit is collected, converted to A/D by the MSP430F149 microcontroller produced by Texas Instruments (Dallas, TX, USA), and communicated to the mobile terminal through Bluetooth to achieve data display. The mobile terminal adopts a smartphone based on the Android system, responsible for receiving pulse wave data, performing waveform playback display, and calculating diastolic and systolic blood pressure values. At the same time, monitoring data are sent to cloud servers through GPRS/Internet networks.

The blood pressure analysis method using Pulse Wave Transit Time (PTT) as a novel, non-invasive, non-compressive, and continuously detectable blood pressure technology is gaining increasing attention.

According to the Moens–Korteweg equation [22], there is a relationship between the Pulse Wave Velocity (*PWV*) and the Young’s modulus of the arterial vessel:

(1) $$PWV=\sqrt{\frac{Eh}{d\rho }}$$

where *E* represents the Young’s modulus of elasticity of the arterial wall, which changes with age, changes in blood composition, and so on. *h* is the thickness of the vessel wall, *d* is the inner diameter of the elastic tube in equilibrium state, and ρ is the blood density.

The relationship between the Young’s modulus of elasticity of the arterial wall and blood pressure, as demonstrated by Hughes et al., is given by:

(2) $$E=E_{0}e^{\gamma P}$$

where *E*_0_ represents the Young’s modulus of elasticity of the arterial wall at zero pressure. *P* represents blood pressure (unit: mmHg, 1 mmHg ≈ 133.322 Pa). *γ* is a parameter measuring vessel characteristics, with a range of values between 0.016 and 0.018 (unit: mmHg^−1^). Substituting Equation (2) into Equation (1), we obtain:

(3) $$PWV=\sqrt{\frac{E_{0}e^{\gamma p}h}{d\rho }}$$

Assuming the length of the arterial vessel through which blood flows between the two measurement positions is L, the relationship between Pulse Wave Velocity (*PWV*) and Pulse Wave Transmission Time (*PTT*) is as follows:

(4) $$PTT=\frac{L}{PWV}$$

By combining Equations (3) and (4), we can obtain:

(5) $$P=\frac{1}{\gamma }\left[\ln\frac{L^{2}\rho d}{gE_{0}}-2\ln PTT\right]$$

Due to the short duration of the measurement signal, the inner diameter *d* and wall thickness of human arterial blood vessels can be ignored, $\ln\frac{L^{2}\rho d}{gE_{0}}$ can be regarded as a constant, and taking the derivative of *PTT* on both sides of the equal sign in the above equation can obtain the functional relationship between blood pressure changes and *PTT*:

(6) $$\frac{dP}{dPTT}=-\frac{2}{\gamma PTT}$$

The above equation can also be expressed as:

(7) $$\Delta P=-\frac{2}{\gamma PTT}\Delta PTT$$

According to Equation (7), when the elasticity of blood vessels remains in a certain state, the conduction time of the pulse waves is directly proportional to the change in blood pressure. For the same individual, the elasticity of their blood vessels does not undergo significant changes in a short period of time. Therefore, by measuring changes in pulse wave conduction time, changes in blood pressure can be estimated, namely:

(8) $$P-P_{0}=b\cdot (PTT-PTT_{0})$$

Simplifying Equation (8) yields:

(9) P=a+b⋅PTT

where *a* and *b* are fitting parameters obtained through polynomial fitting of actual measurements of systolic blood pressure (SBP) and diastolic blood pressure (DBP) with Pulse Wave Transit Time as the independent variable, whose value will vary from individual to individual, but for the same individual, this value is determined in a short period of time.

## 4. Experimental Results and Analysis

### 4.1. Experimental Design and Setup

To verify the performance of the modified polymer, seven different schemes are employed in this paper, including four implementation cases and three comparative ones. Each polymer film obtained from these schemes is cut to a size of 5 cm in length and 3 cm in width. Using a stretching machine, they are uniaxially stretched to a length of 10 cm, resulting in Sample 1 to Sample 7. Sample 1 to sample 4 are modified according to the approach outlined in this paper by adding polyvinyl alcohol and *N*,*N*-dimethylacetamide to modify polyvinylidene fluoride (PVDF), with Sample 4 involving the branching of polyvinyl alcohol using citric acid. On the other hand, Sample 5 to Sample 7 follow different configuration approaches for comparative analysis, highlighting the advantages of the proposed modified polymer. This design aims to comprehensively assess the performance of the modified polymer under various conditions, providing a comprehensive evaluation of its applicability and superiority. The parameters during the production process of each sample are shown in Table 1.

(1) Implementation Case 1

Combine 61 g of polyvinylidene fluoride (PVDF), 19 g of polyvinyl alcohol, and 420 g of *N*,*N*-dimethylacetamide in a container. Stir the mixture uniformly at 60 °C for 4 h to obtain a casting solution. Maintain the temperature of the casting solution, let it stand for 4 min to remove bubbles, then pour the solution onto a glass plate to form a uniform coating with a thickness of 1 mm. Immerse the glass plate with the coating in deionized water at 26 °C. After the coating detaches from the glass plate, keep the coating submerged in deionized water for an additional 50 h to form a film. Remove the film and let it air-dry at room temperature (26 °C) to obtain the polyvinylidene fluoride film. This process results in Sample 1.

(2) Implementation Case 2

Combine 66 g of polyvinylidene fluoride (PVDF), 21 g of polyvinyl alcohol, and 450 g of *N*,*N*-dimethylacetamide in a container. Stir the mixture uniformly at 70 °C for 3 h to obtain a casting solution. Maintain the temperature of the casting solution, let it stand for 4 min to remove bubbles, then pour the solution onto a glass plate to form a uniform coating with a thickness of 1 mm. Immerse the glass plate with the coating in deionized water at 27 °C. After the coating detaches from the glass plate, keep the coating submerged in deionized water for an additional 48 h to form a film. Remove the film and let it air-dry at room temperature (26 °C) to obtain the polyvinylidene fluoride film. This process results in Sample 2.

(3) Implementation Case 3

Combine 71 g of polyvinylidene fluoride (PVDF), 22 g of polyvinyl alcohol, and 500 g of *N*,*N*-dimethylacetamide in a container. Stir the mixture uniformly at 80 °C for 2 h to obtain a casting solution. Maintain the temperature of the casting solution, let it stand for 4 min to remove bubbles, then pour the solution onto a glass plate to form a uniform coating with a thickness of 1 mm. Immerse the glass plate with the coating in deionized water at 28 °C. After the coating detaches from the glass plate, keep the coating submerged in deionized water for an additional 45 h to form a film. Remove the film and let it air-dry at room temperature (26 °C) to obtain the polyvinylidene fluoride film. This process results in Sample 3.

(4) Implementation Case 4

In comparison to Implementation Case 1, the only difference lies in the choice of polyvinyl alcohol. In this case, citric acid-modified polyvinyl alcohol (citric acid-PVA) is used. The preparation method for citric acid-PVA is as follows: Mix 20 g of polyvinyl alcohol with 20 g of citric acid uniformly and react the mixture at 70 °C for 3 h with continuous stirring. After the reaction, cool and crystallize the mixture to obtain citric acid-PVA. The resulting polyvinylidene fluoride film is Sample 4.

(5) Comparison Case 1

In comparison to Implementation Case 1, the key difference in this comparison case is the exclusion of polyvinyl alcohol. The resulting polyvinylidene fluoride film obtained through this method is Sample 5.

(6) Comparison Case 2

In comparison to Implementation Case 1, the key difference in this comparison case is the reduced amount of polyvinyl alcohol, which is 18 g. The resulting polyvinylidene fluoride film obtained through this method is Sample 6.

(7) Comparison Case 3

In comparison to Implementation Case 4, the distinction in this contrast lies in the selection of polyvinyl alcohol, which is replaced with succinic acid–polyvinyl alcohol. The resulting polyvinylidene fluoride film obtained through this method is designated as Sample 7.

### 4.2. Interpretation of Experimental Data

#### 4.2.1. Analysis of Piezoelectric Polymer Properties

**Figure 3:**
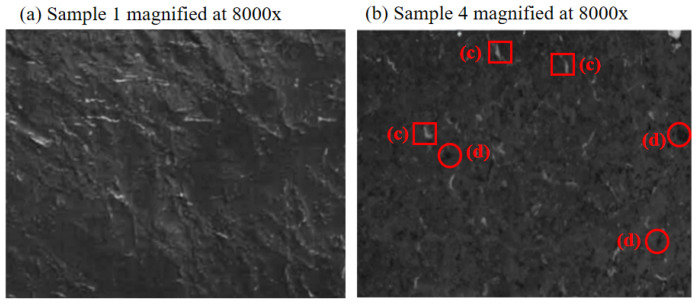
Scanning electron micrographs magnified at 8000×. (**a**) Sample 1; (**b**) Sample 4; (**c**) light-colored fiber cases; (**d**) pores cases.

Scanning electron microscope (SEM) observations are conducted on the surfaces of Sample 1 and Sample 4. The electron micrographs obtained at 8000× magnification are shown in Figure 3a and Figure 3b, respectively.

Figure 3a shows a typical image of polyvinylidene fluoride (PVDF), with a dense and smooth surface. In contrast, Figure 3b introduces some pores and light-colored fibers into the membrane. These pores primarily form during the gelation process, where the two polymers partially separate due to different degrees of shrinkage, creating voids between the two phases. The light-colored fibers may result from the separated polyvinyl alcohol phase. The introduction of these pores and fibers enhances the membrane’s hydrophilicity, water absorption, and breathability, thereby improving its biocompatibility. Therefore, the modified membrane exhibits better water absorption and breathability, making it more biocompatible for use in biological systems.

**Figure 4:**
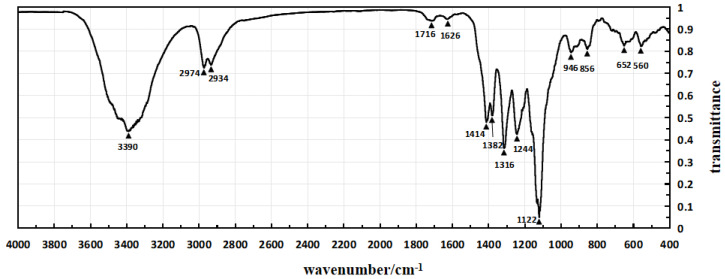
The infrared spectrum of Sample 1.

**Figure 5:**
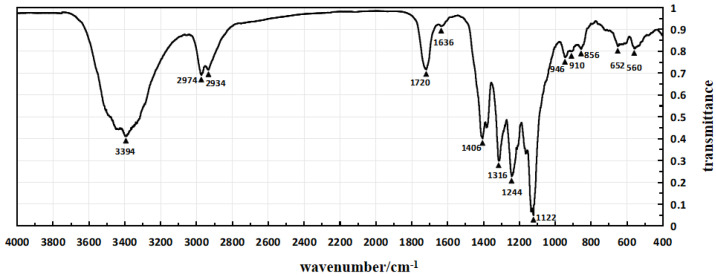
The infrared spectrum of Sample 4.

**Figure 6:**
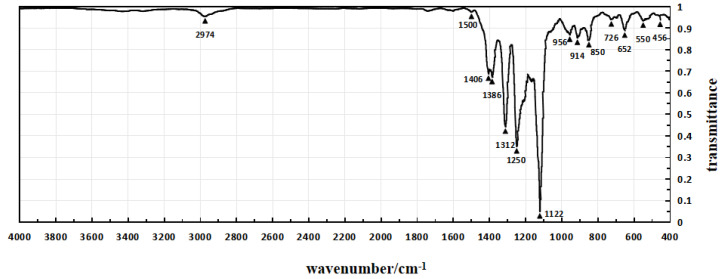
The infrared spectrum of Sample 5.

The infrared spectra of Sample 1, Sample 4, and Sample 5 are shown in Figure 4, Figure 5 and Figure 6, respectively, which are obtained through testing with a Fourier transform infrared spectrometer.

In the infrared spectrum shown in Figure 4, the absorption peak at 3390 cm^−1^ indicates the stretching vibration of hydroxyl groups (O-H), while the peak at 2934 cm^−1^ corresponds to the stretching vibration of methylene groups. The absorption peak at 1716 cm^−1^ represents the second harmonic of PVDF vibration, and the peak at 1626 cm^−1^ corresponds to the in-plane bending vibration of residual water-OH. The enhanced absorption peak at 1414 cm^−1^ is attributed to the in-plane bending vibration of alcohol-COH, and it also exhibits characteristic absorption peaks of -OH in polyvinyl alcohol.

In the infrared spectrum shown in Figure 5, the absorption peak at 1720 cm^−1^ corresponds to the stretching vibration of ester and carboxylic acid C=O, indicating the introduction of citric acid. However, there are minimal changes in other absorption peaks. Additionally, the variations in other absorption peaks are relatively small. These infrared spectra provide detailed structural information about the introduced chemical components in the experiment, aiding in confirming the success of the modification process and the characteristics of the resulting product.

In the infrared spectrum shown in Figure 5, the frequencies and intensities of various absorption peaks provide detailed information about the molecular structure. The peak at 2974 cm^−1^ represents the stretching vibration of the -CF2-CH2- methyl group, which shifts to higher frequencies due to the electronegativity of the fluorine atoms. The peaks at 1406 and 1386 cm^−1^ correspond to the bending vibration of the methyl group, while the peak at 1312 cm^−1^ represents the out-of-plane wagging vibration of the methyl group. The broad absorption peaks at 1250 and 1122 cm^−1^ indicate the antisymmetric and symmetric stretching vibrations of CF2. The peaks at 956, 914, and 850 cm^−1^ are characteristic absorption peaks of the crystalline phase of PVDF, with the peak at 850 cm^−1^ corresponding to the bending vibration of C-C-C. The peaks at 652 and 550 cm^−1^ represent the antisymmetric and symmetric bending vibrations of CF2. The appearance and positions of these characteristic absorption peaks provide fingerprints for the molecular structure of the modified polymer, aiding in confirming the introduction of structural units and the ordered arrangement of molecules during the modification process.

The piezoelectric properties of the 7 samples are tested using the piezoelectric pulse method, and the calculated piezoelectric constants and dielectric constants of the samples are shown in Table 2. Analysis of the data in Table 2 reveals that the piezoelectric and dielectric constants of the polyvinylidene fluoride (PVDF) films corresponding to Sample 1 to Sample 4 are significantly higher than those of Sample 5 to Sample 7. This indicates that the modified piezoelectric polymer developed in this paper exhibits superior piezoelectric performance. This finding underscores the superiority of Sample 1 to Sample 4 in terms of piezoelectric performance, providing strong experimental support for the improvement and expansion of the applications of piezoelectric polymers.

#### 4.2.2. Analysis of Intelligent Blood Pressure Monitoring Performance

**Figure 7:**
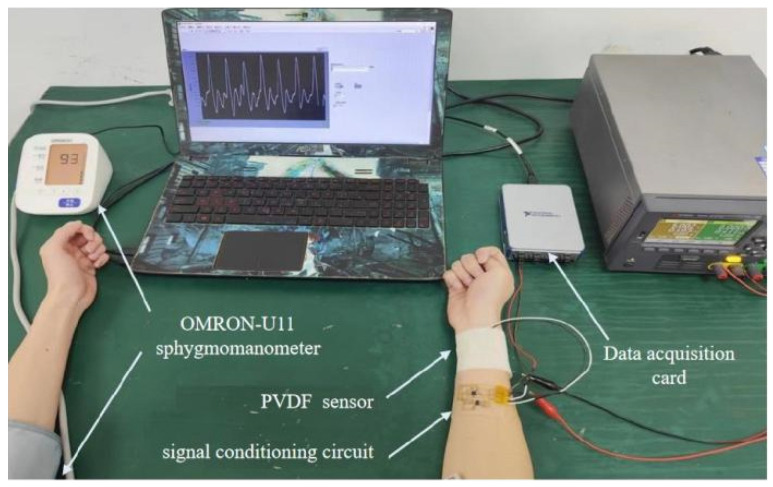
Pulse collection environment.

To verify the accuracy of the fitting parameters, a comparative experiment is conducted. One participant underwent blood pressure measurements five times during the same time period, and the obtained systolic and diastolic pressures are compared with the measurements from a conventional electronic blood pressure monitor (OMRON-U11 model). The process of collecting pulse signals is shown in Figure 7. First, attach the PVDF pressure sensor to the wrist of the right hand, and then tie the cuff of the Omron electronic blood pressure monitor to the left upper arm of the volunteer, using its measurement result as the standard value.

By analyzing the experimental data, the average relative error, which refers to the ratio of absolute error to the measured value or the ratio of absolute error to the average of multiple measurements, is calculated and the results are compiled in Table 3. The average relative error is an important indicator for evaluating the simulation effect of a model. The purpose of this comparative experiment is to assess the accuracy of the fitting parameters in a real blood pressure monitoring scenario. By comparing with the electronic blood pressure monitor, it provides a comprehensive evaluation of the performance and accuracy of the fitting model.

In the experiment, the 7 samples are individually secured on medical tape, reinforced with hot-melt adhesive, and connected to a voltmeter via test leads. A force gauge is used to calibrate the linear relationship between the force applied to the film and the resulting voltage. The film samples attached with medical tape are wrapped around the subject’s upper arm, and a stethoscope is placed. By gradually tightening the medical tape, the Korotkoff sound method is used to read the voltage corresponding to diastolic and systolic pressures. These readings are then converted into corresponding pressure values using a linear equation, resulting in blood pressure measurement results in the format “diastolic pressure/systolic pressure”, with units in mmHg. It is important to note that this method is suitable for single-measurement comparisons, while in industrial production, calibration is typically performed before products leave the factory, and continuous monitoring relies on automated electronic devices and signal processing systems. Following blood pressure measurement conventions, blood pressure values and averages are rounded to the nearest whole number, while standard deviation and relative error are rounded to one decimal place. Each set of samples was tested 5 times, and the test results are shown in Table 4.

Analysis of the data in Table 3 and Table 4 reveals that among all the samples, Sample 1 exhibits the lowest standard deviation and relative error compared to the electronic sphygmomanometer, showing results closely aligned with the mercury sphygmomanometer. Additionally, the standard deviation and relative error of Sample 1 to Sample 4 are lower than those of Sample 5 to Sample 7. This indicates that the use of the piezoelectric polymer developed in this paper allows for more accurate blood pressure measurements.

**Figure 8:**
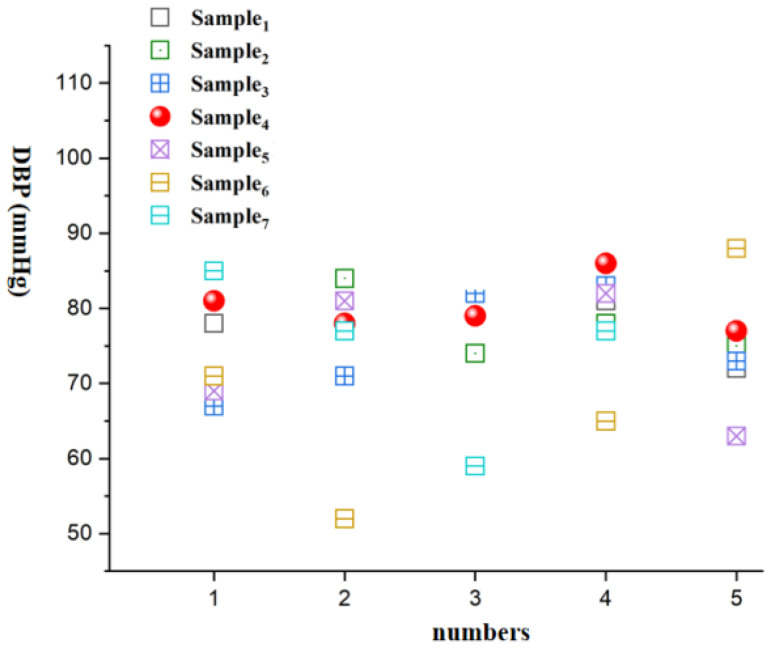
DBP vs. numbers.

**Figure 9:**
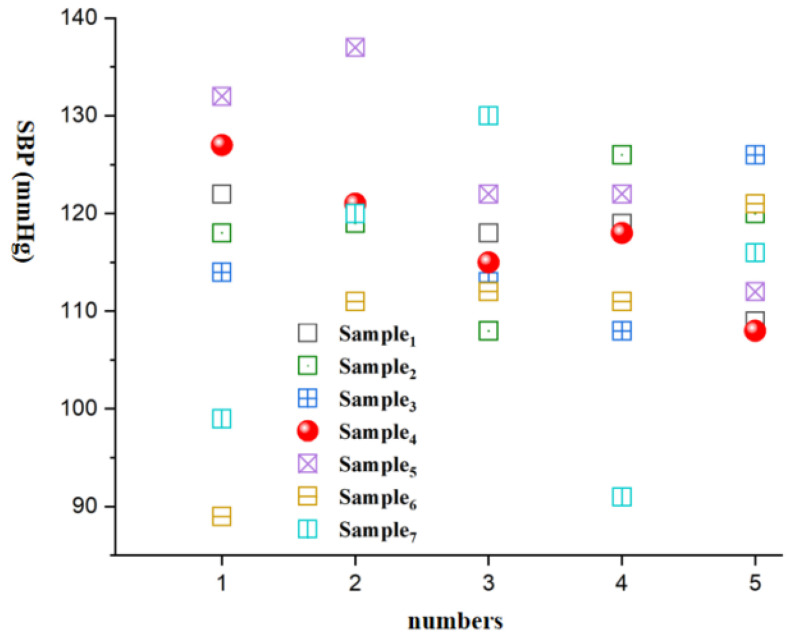
SBP vs. numbers.

**Figure 10:**
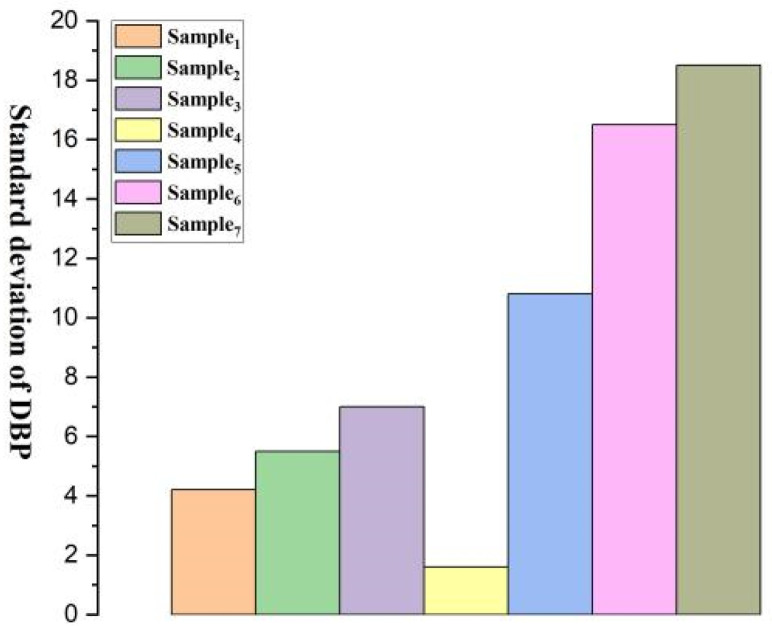
Standard deviation of DBP for Samples 1–7.

**Figure 11:**
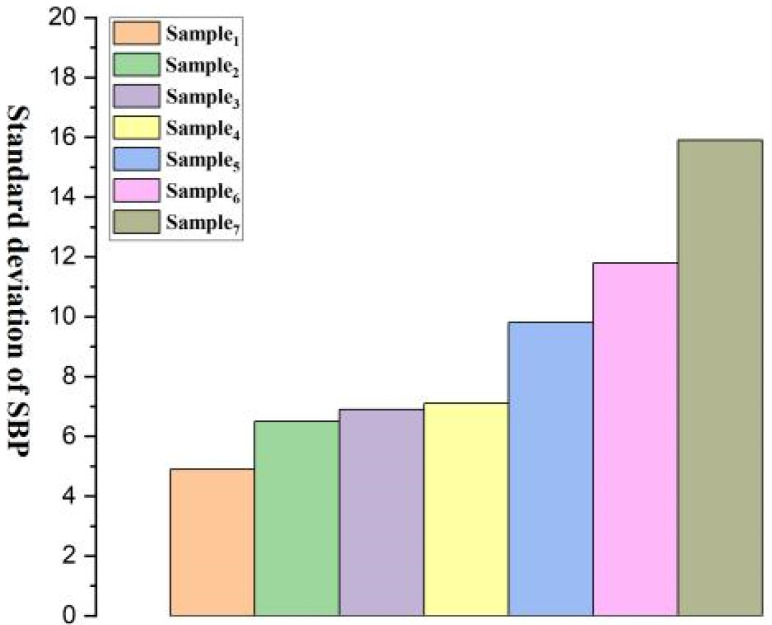
Standard deviation of SBP for Samples 1–7.

**Figure 12:**
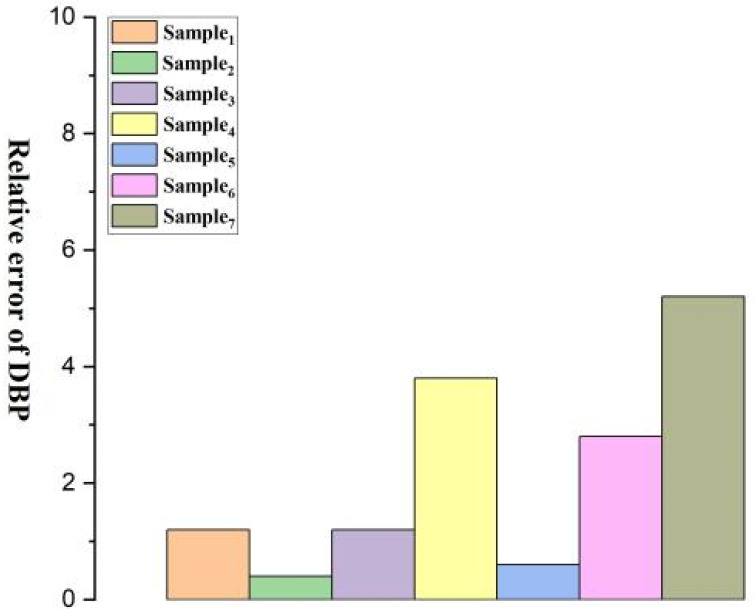
Relative error of DBP for Samples 1–7.

**Figure 13:**
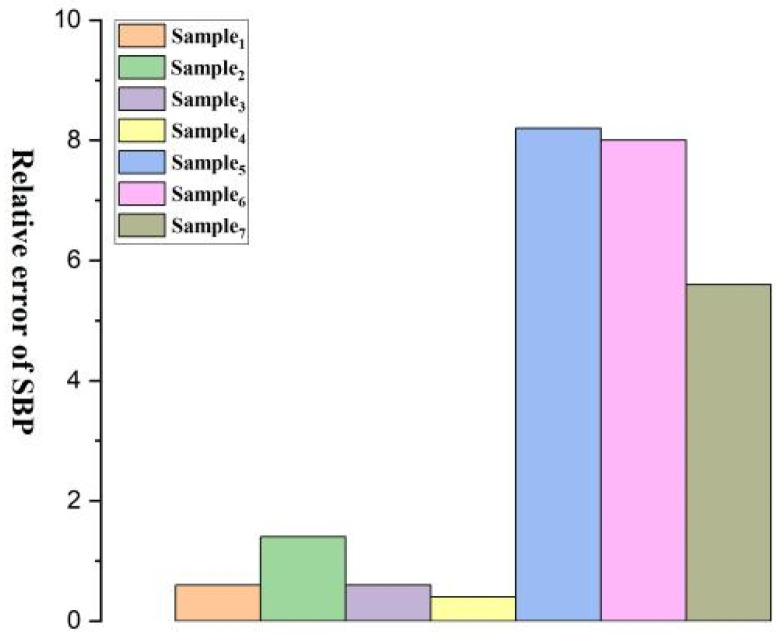
Relative error of SBP for Samples 1–7.

Figure 8, Figure 9, Figure 10, Figure 11, Figure 12 and Figure 13 show five blood pressure measurements of the seven samples.

As shown in the figures, the standard deviation and relative error of Samples 1 to 4 are lower than those of Samples 5 to 7, indicating that the use of the piezoelectric polymer developed in this paper can improve the accuracy of blood pressure measurement.

Sample 4 possesses a larger dielectric constant, resulting in relatively more stable test outcomes, although the relative error is slightly higher than that of Sample 1. However, in the case of Sample 7, where succinic acid is used instead of citric acid for grafting, the results prove that citric acid is a more suitable choice. Compared to Sample 4, the piezoelectric coefficient and dielectric constant of Sample 7 significantly decrease. This is attributed to the poor compatibility between succinic acid-grafted polyvinyl alcohol (PVA) and polyvinylidene fluoride (PVDF). Succinic acid, after esterification with polyvinyl alcohol, leaves only one free carboxyl group, whereas citric acid, after esterification, leaves two free carboxyl groups and one hydroxyl group. The free hydroxyl group facilitates better integration of citric acid branches into the polyvinyl alcohol molecular chain, contributing to an enhanced dielectric constant. The larger number of free carboxyl groups in citric acid provides greater polarity. The research findings underscore the importance of improving the performance of piezoelectric polymers, offering valuable guidance for the development of more accurate blood pressure monitoring systems.

Infrared spectroscopy tests were taken for Sample 1, Sample 4, and Sample 5, and the test results are presented in Table 5.

Analysis of the data in Table 5 reveals that Sample 1’s piezoelectric polymer has successfully introduced polyvinyl alcohol molecules between polyvinylidene fluoride molecules. Meanwhile, Sample 4 exhibits absorption peaks of carboxyl and ester groups, confirming the successful branching of polyvinyl alcohol by citric acid and its successful introduction into the piezoelectric polymer through modification treatment. Therefore, the experimental results confirm the successful modification of Sample 1 and Sample 4 at the molecular level, providing strong experimental support for the further application of piezoelectric polymers. This indicates a significant achievement in the introduction of polyvinyl alcohol and branching treatment, laying a substantial foundation for expanding the functionality and performance of piezoelectric polymers.

## 5. Conclusions

This paper successfully modifies polyvinylidene fluoride by introducing polyvinyl alcohol and *N*,*N*-dimethylacetamide, resulting in a novel piezoelectric polymer with excellent piezoelectric performance and electromechanical coupling characteristics. The introduction of *N*,*N*-dimethylacetamide promotes the formation of the film’s β-phase, enhancing the piezoelectric coefficient and sensitivity, simplifying the processing steps, and reducing the glass transition temperature, thereby improving flexibility and wearability. Citric acid branching increases the dielectric constant and charge storage capability of the piezoelectric polymer, enhancing its stability and reliability while also accelerating the degradation rate of polyvinylidene fluoride, thus enhancing environmental friendliness and sustainability. These improvements enable the material to exhibit increased sensitivity in blood pressure monitoring systems, thereby enhancing the accuracy of blood pressure monitoring. This paper provides an innovative material solution for future intelligent health monitoring systems, contributing significantly to advancements in medical technology.

## Figures and Tables

**Table 1:** Process conditions for Samples 1–7.

	Implementation 1 Sample 1	Implementation 2 Sample 2	Implementation 3 Sample3	Implementation 4 Sample 4	Comparison 1 Sample 5	Comparison 2 Sample 6	Comparison 3 Sample 7
PVDF	61 g	66 g	71 g	---	61 g	61 g	---
citric acid-PVA	---	---	---	61 g	---	---	61 g
polyvinyl alcohol	19 g	21 g	22 g	19 g	---	18 g	---
succinic acid–polyvinyl alcohol	---	---	---	---	---	---	19 g
*N*,*N*-dimethylacetamide	420 g	450 g	500 g	420 g	420 g	420 g	420 g
stirring temperature	60 °C	70 °C	80 °C	60 °C	60 °C	60 °C	60 °C
stirring time	4 h	3 h	2 h	4 h	4 h	4 h	4 h
deionization temperature	26 °C	27 °C	28 °C	26 °C	26 °C	26 °C	26 °C
deionization time	50 h	48 h	45 h	50 h	50 h	50 h	50 h
characteristics	dense and smooth surface			more biocompatible			

**Table 2:** Piezoelectric constants and dielectric constants of different samples.

Sample Type	Piezoelectric Coefficient d33 (pC/N)	Dielectric Constant (Relative to Vacuum)
Sample 1	−41.2	13.4
Sample 2	−40.3	13.1
Sample 3	−38.8	12.8
Sample 4	−40.9	14.2
Sample 5	−33.0	6.7
Sample 6	−34.2	7.1
Sample 7	−32.1	7.0

**Table 3:** Obtained diastolic/systolic pressures.

Number of Measurements	1	2	3	4	5	Mean Value	Standard Deviation
Electronic Blood Pressure Monitor	77/119	73/113	71/114	81/123	80/115	76/117	4.3/4.2

**Table 4:** Blood pressure monitoring results based on different samples.

Number of Measurements	Sample 1	Sample2	Sample 3	Sample 4	Sample 5	Sample 6	Sample 7
1	78/122	69/118	67/114	81/127	69/132	71/89	85/99
2	71/119	84/119	71/120	78/121	81/137	52/111	77/120
3	74/118	74/108	82/113	79/115	90/122	92/112	59/130
4	81/119	78/126	83/108	86/118	82/122	65/111	77/91
5	72/109	75/120	73/126	77/108	63/112	88/121	110/116
Mean Value	75/117	76/118	75/116	79/118	77/125	74/109	82/111
Standard Deviation	4.2/4.9	5.5/6.5	7.0/6.9	1.6/7.1	10.8/9.8	16.5/11.8	18.5/15.9
Relative Error	1.2/0.6	0.4/1.4	1.2/0.6	3.8/0.4	0.6/8.2	2.8/8.0	5.2/5.6

**Table 5:** The infrared spectroscopy test results for Sample 1, Sample 4, and Sample 5.

Sample 1		Sample 4		Sample 5	
Peak Wavenumber (cm^−1^)	Transmittance	Peak Wavenumber (cm^−1^)	Transmittance	Peak Wavenumber (cm^−1^)	Transmittance
560	82%	560	81%	550	93%
652	83%	652	82%	652	89%
856	81%	856	81%	700	95%
946	80%	910	80%	726	94%
1122	5%	946	77%	850	84%
1244	43%	1122	5%	914	85%
1316	36%	1244	23%	956	87%
1382	51%	1316	30%	1122	5%
1414	48%	1406	40%	1168	65%
1626	95%	1636	91%	1250	35%
1716	94%	1720	72%	1312	44%
2934	74%	2934	72%	1386	67%
2974	73%	2974	69%	1406	69%
3390	44%	3394	41%	2974	95%

## Data Availability

All the datasets used in this manuscript are publicly available datasets already in the public domain.
